# Validating Habitual and Goal-Directed Decision-Making Performance Online in Healthy Older Adults

**DOI:** 10.3389/fnagi.2021.702810

**Published:** 2021-06-29

**Authors:** Kaori L. Ito, Laura Cao, Renee Reinberg, Brenton Keller, John Monterosso, Nicolas Schweighofer, Sook-Lei Liew

**Affiliations:** ^1^Neural Plasticity and Neurorehabilitation Laboratory, Department of Occupational Science and Occupational Therapy, University of Southern California, Los Angeles, CA, United States; ^2^Computational Neuro-Rehabilitation Laboratory, Department of Biokinesiology and Physical Therapy, University of Southern California, Los Angeles, CA, United States; ^3^Department of Gerontology, University of Southern California, Los Angeles, CA, United States; ^4^Department of Psychology, University of Southern California, Los Angeles, CA, United States

**Keywords:** validating, decision-making, goal-directed, habitual, aging, older adults, online, reinforcement learning

## Abstract

Everyday decision-making is supported by a dual-system of control comprised of parallel goal-directed and habitual systems. Over the past decade, the two-stage Markov decision task has become popularized for its ability to dissociate between goal-directed and habitual decision-making. While a handful of studies have implemented decision-making tasks online, only one study has validated the task by comparing in-person and web-based performance on the two-stage task in children and young adults. To date, no study has validated the dissociation of goal-directed and habitual behaviors in older adults online. Here, we implemented and validated a web-based version of the two-stage Markov task using parameter simulation and recovery and compared behavioral results from online and in-person participation on the two-stage task in both young and healthy older adults. We found no differences in estimated free parameters between online and in-person participation on the two-stage task. Further, we replicate previous findings that young adults are more goal-directed than older adults both in-person and online. Overall, this work demonstrates that the implementation and use of the two-stage Markov decision task for remote participation is feasible in the older adult demographic, which would allow for the study of decision-making with larger and more diverse samples.

## Introduction

Since its conception, the two-stage Markov decision task has been widely used across many studies to investigate decision-making behavior. One reason for its widespread popularity is that the two-stage task allows the dissociation of model-based from model-free reinforcement learning (RL) algorithms, which traditionally have been considered the computational proxy of goal-directed and habitual decision making, respectively (Gläscher et al., 2010; Redgrave et al., 2010; Daw et al., 2011). Goal-directed decision making is cognitively demanding and is slow and deliberate. In goal-directed or model-based decision making, the decision-maker keeps track of which actions are likely to lead to rewards and updates their internal model based on changes in reward values associated with an action, requiring the use of working memory (Balleine and O’doherty, 2010). In contrast, habitual decision making is reflexive and reflects a simple stimulus-response association. The model-free or habitual decision-maker simply learns which actions lead to rewards by experiencing the consequences of its actions (Sutton and Barto, 2018). In habitual decision-making, the association between the stimulus and response persists even after a reward is devalued.

Previous research has shown that we typically use both strategies in parallel, but there is some individual variability in the propensity toward one decision making strategy over another. Further, the balance between habitual and goal-directed strategies has been shown to shift with various factors: with age, greater stress, and compulsivity, the balance shifts toward more habitual decision-making strategies, whereas greater working memory capacity has been associated with more goal-directed strategy and in fact protects goal-directed strategies from the effects of stress (Eppinger et al., 2013; Otto et al., 2013; de Wit et al., 2014; Voon et al., 2014; Linnebank et al., 2018). With the exception of working memory, however, it is unknown whether these factors interact with age in shifting the decision-making balance. Our goal was to examine how aging interacts with psychosocial factors in shifting decision-making processes. We began data collection in early 2020, however, with the onset of the SARS-CoV-2 coronavirus (COVID-19) pandemic, all in-person research was halted due to social distancing requirements and safety precautions, and we subsequently transitioned our study online. However, this necessitated that we validate that performance on the two-stage task online was comparable to that in-lab.

While the original two-stage Markov tasks were conducted in-person (Gläscher et al., 2010; Daw et al., 2011), a handful of studies using the task have been conducted online (Gillan et al., 2015; Nussenbaum et al., 2020). In general, web-based studies benefit from convenience (i.e., eliminating travel) and larger samples (Berinsky et al., 2012). Data collected through online experiments has also been shown to be comparable to the quality of data collected in person (Crump et al., 2013). However, to our knowledge, only Nussenbaum et al. (2020) has compared online to in-person study participation on the two-stage task in particular on children, adolescents, and young adults, but this has not yet been validated in the older adult population.

Given that remote study participation requires some measure of technological proficiency on the part of the participant, examining whether decision-making data collected online is comparable to in-person data in the older adult population is especially important as older adults tend to use the internet less than younger age groups and require more time to learn computer programs (White et al., 1999; Adams et al., 2005). Further, because the two-stage task can be quite lengthy (lasting 40 min to 1 h) and involves making many repeated choices, it requires concentration, which may be more difficult outside of the quiet laboratory setting with an experimenter physically present. Thus, in this study, we implemented and validated a web-based version of the two-stage Markov task using parameter simulation and recovery and compared behavioral results from online and in-person participation on the two-stage task in both young and older adults. As previous in-person studies have shown that age shifts the balance between goal-directed and habitual decision making (Eppinger et al., 2013), here we also aimed to replicate this finding and examine whether the same pattern holds with data collected online.

## Materials and Methods

### Participants

A total of 42 healthy young adults (YA) and 41 healthy older adults (OA) participated in the study. Of these, 12 YA and 11 OA participated in the study in-person prior to the onset of the COVID-19 pandemic, and 30 YA and 30 OA participated in the study online. Participants were recruited through convenience sampling and through social media. All participants were recruited from the United States only, and had to score ≥ 19 on the telephone-Montreal Cognitive Assessment (t-MoCA). Young adult participants had to be between 18 and 49 years of age; older adult participants had to be between 50 and 80 years of age. Individuals were excluded if they were left-handed or ambidextrous, did not have normal or corrected-to-normal visual acuity, did not speak English proficiently, or if they had any history of neurological conditions. Informed consent was obtained from all participants in accordance with procedures approved by the University of Southern California Institutional Review Board (IRB), and participants received monetary compensation, either in cash (in-person participants) or in the form of electronic gift cards (online participants).

### Task Description and Implementation

The task was implemented in MATLAB R2019b (MathWorks Inc., MA, United States) for both the in-person and online participants. Due to the difficult nature of the traditional two-stage Markov task, we used a modified version, in which we included only one decision per trial, rather than two, for simplicity (similar to that presented in Gillan et al., 2015). Participants were given training and a quiz prior to participating in the task. If they missed more than one question on the quiz, they were sent back to repeat the training again (see Supplementary Material).

There were 201 trials in this task, divided up into three blocks of 67 trials each. We implemented a 1-min break between each block, during which we gave a reminder that the goal of the task was to earn as much money as possible, up to $10.

**FIGURE 1:**
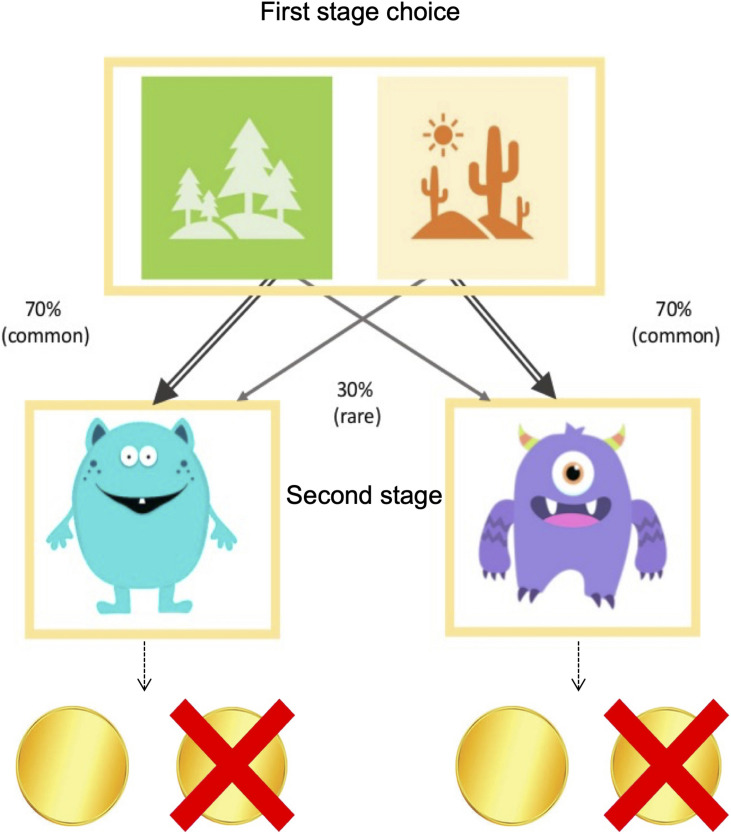
Two-stage Markov Decision Task. Participants were given a choice between one of two start states on each trial, a forest and a desert. One location more commonly (70%) led to one of the second-stage states (the blue and purple cartoons), and rarely (30%) led to the other. Each second-stage state was associated with slowly changing reward probabilities.

Each trial consisted of two subsequent stages followed by a reward outcome state (Figure 1 and see Supplementary Video 1). At the start of each trial, a choice between two images is presented in the first stage (a forest and desert). Each image is probabilistically associated with two possible subsequent states in the second stage (a blue or a purple alien cartoon). Specifically, selecting the forest will result in revealing the blue cartoon 70% of the time, and the purple cartoon 30% of the time, and selecting the desert will result in revealing the purple cartoon 70% of the time, and the blue cartoon 30% of the time. Each second stage state (i.e., the blue and the purple cartoon) is associated with a slowly changing reward probability of either earning a coin (worth 5 cents) or earning nothing, according to Gaussian random walks to incentivize continued learning. In the original version of this task, a second choice is presented at this second stage, however, we eliminated the choice at the second stage to simplify the task, similar to the version used in Gillan et al. (2015).

The logic of the task is such that a habitual or model-free decision-maker, would, if rewarded, be more likely to stay with the same first-stage choice (i.e., forest or desert) on the next trial even if the first-stage choice led to a second-stage cartoon to which it was less commonly associated (i.e., 30%). On the other hand, the goal-directed or model-based decision-maker would exhibit a *decreased* tendency to repeat the same option because the goal-directed decision-maker would take the task’s 70–30 transition structure into account and choose the first-stage option that was not originally chosen.

### Additional Online Task Specifications

The online task was administered through the use of Google Chrome Remote Desktop^1^ to avoid requiring participants to install applications. To support online data collection, participants were required to have a home computer with high-speed internet (≥60 mbps, as determined by an internet speed test; see section “Discussion”). The online version of the task was implemented as described above, with the exception of adding in an additional measure to prevent online participants from making random responses on the task as the experimenter could not be physically present to monitor responses. The task was designed to give a warning if participants made consecutive key presses, alternating key presses, chose the same location, or missed making choices for above a certain number of trials. After the first warning, participants would have one more chance at the task before the study was aborted. To ensure the flow and quality of data collection, the researcher called the participant to walk through the set-up of Chrome Remote Desktop and stayed on the line (muted) until the participant completed the entire task.

### Statistical Analysis

#### Participant Demographics

Participant demographics between in-person and online groups in both young and older adults were compared using two-sample *t*-tests for continuous data and Chi-square for count data.

#### Simulation and Validation of Model Implementation

A standard hybrid reinforcement learning model originally proposed by Daw et al. (2011) was used to fit choice behavior on the two-stage task. The hybrid model combines the model-free (habitual) SARSA (λ) algorithm (Rummery and Niranjan, 1994) with model-based (goal-directed) learning (see Supplementary Material) to analyze choice behavior. In brief, the standard hybrid model includes five free parameters: weight, α, β, λ, and perseveration. Weight represents each individual’s propensity toward model-based (goal-directed) and model-free (habitual) behavior, and ranges from 0 to 1, where values closer to 0 represent more model-free behavior, and values closer to 1 represent more model-based behavior. α controls how much the parameters are adjusted with respect to the previous trial, ranging from 0 to 1, where 0 represents no learning and 1 represents responding solely to the last trial. β controls the stochasticity of choice, ranging from β = 0 for completely random responding and β = 20 for deterministically choosing responses. λ represents short term memory ranging from 0 to 1, where a higher λ represents a longer lasting memory trace, e.g., the decision is weighted more by the first stage cartoon, whereas λ = 0 represents a case where only the second stage location plays a role in the choice. Finally, perseveration represents the “stickiness” of the last choice that was made, with values ranging from −1 to 1, where a lower value represents more switching, and a higher value represents more repeated choices.

We first simulated decisions for pure model-free and model-based agents in MATLAB, as well as decisions by the hybrid reinforcement learning model. For each model, we randomly drew 100 samples from a normal distribution with mean μ and standard deviation 0.2, where μ is the transformed parameter value as reported in Daw et al. (2011) and da Silva and Hare (2020) (see Table 2 for input parameter values).

**TABLE 1:** Demographics.

	In-person OA (*n* = 11)	Online OA (*n* = 30)	In-person YA (*n* = 12)	Online YA (*n* = 30)
Age mean ± SD, range	62.81 ± 9 (51–76)	60.70 ± 7 (50–73)	24.17 ± 3 (19–29)	31.70 ± 6 (20–47)
	*t* = 0.71, *p* = 0.49		*t* = -5.17, *p* < 0.0001	
Gender	5 Male, 6 Female	7 Male, 23 Female	1 Male, 11 Female	8 Male, 22 Female
	*χ*^2^ = 0.98, *p* = 0.32		*χ*^2^ = 0.37, *p* = 0.80	
Education	6 Graduate degree, 3 Bachelor’s degree, 1 Associate degree, 1 Some college/no degree	13 Graduate degree, 13 Bachelor’s degree, 1 Associate degree, 3 Some college/no degree	3 Graduate degree, 7 Bachelor’s degree, 2 Some college/no degree	20 Graduate degree, 8 Bachelor’s degree 2 Some college/no degree
	χ^2^ = 1.30, *p* = 0.73		χ^2^ = 7.51, *p* = 0.06	
Marital status	4 Married or domestic partnership, 3 Divorced, 1 Widowed, 3 Single, never married	21 Married or domestic partnership, 4 Divorced, 2 Widowed, 2 Single, never married, 1 Separated	12 Single, never married	11 Married or domestic partnership, 18 Single, never married, 1 Divorced
	χ^2^ = 5.64, *p* = 0.23		χ^2^ = 7.70, *p* = 0.02	
Race	6 White, 4 Asian, 1 from multiple races	13 White, 14 Asian, 3 Black or African-American	1 White, 2 Black or African-American, 6 Asian, 2 Hispanic or Latino, 1 N/A	5 White, 25 Asian
	χ^2^ = 4.24, *p* = 0.23		χ^2^ = 14.21, *p* < 0.001	

*Participant demographics for each group. OA, older adults; YA, young adults.*

**TABLE 2:** Generated (input) and estimated parameters for simulations.

	α (0–1)	β (0–20)	Weight (0–1)	λ (0–1)	Perseveration (−1 to 1)
Daw et al. (2011)	Input: 0.54	Input: 5.24	Input: 0.39	Input: 0.57	Input: 0.12
	Estimated: 0.55	Estimated: 5.22	Estimated: 0.36	Estimated: 0.54	Estimated: 0.11
	Difference: 0.01	Difference: 0.02	Difference: 0.03	Difference: 0.03	Difference: 0.01
Pure Model Based (da Silva and Hare, 2020)	Input: 0.55	Input: 5.18	Input: 1	Input: 0.49	Input: 0
	Estimated: 0.50	Estimated: 4.04	Estimated: 0.83	Estimated: 0.49	Estimated: -0.05
	Difference: 0.05	Difference: 1.14	Difference: 0.17	Difference: <0.001	Difference: 0.05
Pure Model Free (da Silva and Hare, 2020)	Input: 0.49	Input: 5.16	Input: 0	Input: 0.49	Input: 0
	Estimated: 0.49	Estimated: 6.24	Estimated: 0.22	Estimated: 0.49	Estimated: 0.3
	Difference: <0.01	Difference: 1.07	Difference: 0.22	Difference: <0.01	Difference: 0.3
Hybrid (da Silva and Hare, 2020)	Input: 0.51	Input: 5.12	Input: 0.51	Input: 0.50	Input: 0.01
	Estimated: 0.54	Estimated: 4.29	Estimated: 0.45	Estimated: 0.52	Estimated: 0.13
	Difference: 0.03	Difference: 0.83	Difference: 0.06	Difference: 0.02	Difference: 0.12

*Generated (input) and estimated parameters for simulations. Estimated and difference between estimated and input are based on the mean of 100 simulations.*

The simulated choices were then fit to a mixed logistic regression model using the lme4 package in the R programming language, version 4.0.3^2^. Choice (coded as *switch* = 0 and *stay* = 1, relative to the previous choice) was the dependent variable, and the independent variables were the reward received on the previous trial, a binary variable indicating whether the previous trial’s transition was common or rare, and the interaction of the two. Following this, we fit the choice data to the hybrid model using the Stan modeling library in R to obtain parameter estimates for validation.

#### Behavioral Data Analysis

For each group, we conducted a mixed logistic regression analysis on the behavioral data using the model specified in section “Simulation and Validation of Model Implementation” to examine trial-by-trial adjustments in choice preferences for each group during the task. Specifically, the specification for the regression was choice - reward ^∗^ transition + (1 + reward × transition | subject).

Next, the observed sequences of choices and rewards were used to estimate free parameters of the hybrid model (α, β, λ, weight, and perseveration; refer to section “Simulation and Validation of Model Implementation”) for each individual participant, using Markov chain Monte Carlo sampling for Bayesian modeling, implemented in Stan. The posterior median was used as the parameter estimate for each parameter.

Our primary goal was to compare performance between the two participation mediums (in-person and online) on the two-stage task in both young and older adults. However, as mentioned above, previous studies have also shown that age shifts the balance between goal-directed and habitual decision-making. Based on this, we hypothesized that the older adult group would have a lower weight parameter, indicating more habitual decision-making, than the young adult group. Thus, we conducted a two-way ANOVA with age group and participation setting as factors, to examine the effects of each and their interaction effect on the weight parameter. We also performed two-way ANOVAs on the other parameters to examine the effects of study participation setting as well as age on each of the parameters.

Because the in-person groups were relatively small, we performed a follow up analysis of covariance (ANCOVA) on each of the estimated parameters, combining the two age groups into a single group by using age as a continuous variable. We first tested for an interaction effect between age and participation setting, and then re-ran the ANCOVA without the interaction term with the following specification in R: parameter - age + factor (participation setting).

## Results

### Participant Demographics

Participant demographics are reported in Table 1. There were no significant differences in age, marital status, gender distribution, racial distribution, and level of education between the two OA groups (Table 1). There was no significant difference in gender distribution between the YA in-person and online groups. However, age, racial distribution, education, and marital status were significantly different between the two YA groups. Specifically, the online group was overall older (*p* < 0.0001), more diverse in marital status (*p* = 0.02), and more educated (*p* = 0.06) than the in-person group while the in-person group was more diverse in racial distribution (*p* < 0.001; Table 1).

### Simulation and Validation Results

**FIGURE 2:**
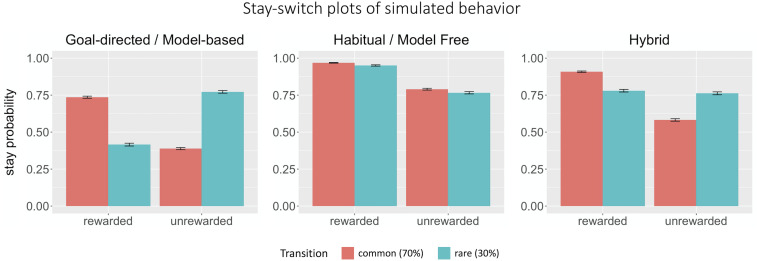
Stay-switch plots of simulated behavior. Graphs depicting purely model-based (goal-directed), purely model-free (habitual), and hybrid behaviors. Purely model-based behavior is predicted by an interaction between reward and transition, whereas purely model-free behavior is predicted solely by reinforcement history. Hybrid behavior represents a mix of model-based and model-free behavior.

Consistent with previous studies (Daw et al., 2011), our simulation results showed that a model-free, or habitual, strategy predicts only a main effect of reward (β*_*reward*_* = 1.90, *p* < 0.0001), while a model-based or goal-directed strategy predicts an interaction effect between reward and transition (β*_*interaction*_* = 3.07, *p* < 0.0001; Figure 2). Estimated parameters closely matched all parameters except perseveration used to generate simulated data in the pure model-free, model-based, and hybrid cases (Table 2).

### Trial-by-Trial Adjustments in Choice Preferences

**FIGURE 3:**
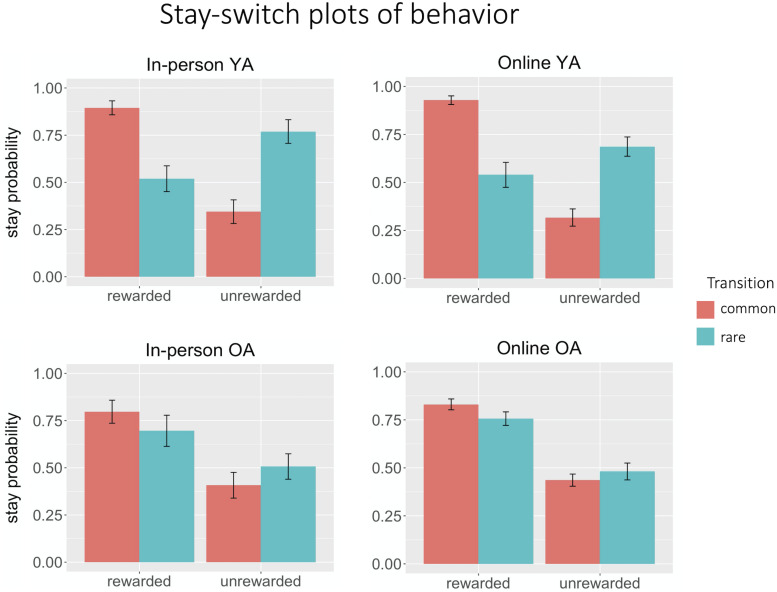
Stay-switch plots of behavior. Both in-person (*n* = 12) and online (*n* = 30) young adult groups exhibited hybrid behavior, that is, a mix of model-based and model-free behavior. In both in-person (*n* = 11) and online (*n* = 30) older adult groups, behavior was more characteristic of habitual performance, with a strong effect of reward but an attenuated effect of transition.

For young adults, both the main effect of reward (in-person β*_*reward*_* = 0.56; online β*_*reward*_* = 0.92; *p* < 0.0001) and the reward × transition interaction (in-person β*_*interaction*_* = 1.37; online β*_*interaction*_* = 1.40; *p* < 0.001) were significant in the in-person and online groups, suggesting that they used a mixture of goal-directed and habitual strategies (Figure 3).

For both older adult groups, the main effect of reward was also significant (Figure 3; in-person β*_*reward*_* = 0.94; online β*_*reward*_* = 1.10; *p* < 0.001), but the reward × transition interaction was only significant for the online older adult group (β*_*interaction*_* = 0.14, *p* = 0.03).

### Effects of Participation Medium and Age

**FIGURE 4:**
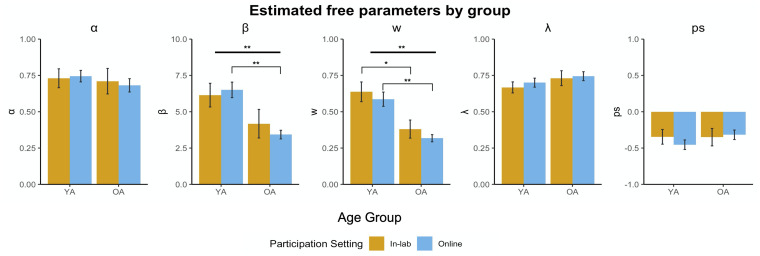
Estimated free parameters by group. We performed 2-way ANOVAs assessing main effects of age group (young adults vs. older adults) and participation medium (in-person vs. online) for each parameter. There were no main effects of participation medium, but we found main effects of age for weight (w) and β. Bolded lines represent significant main effects, brackets represent significant pairwise comparisons (**p < 0.001, *p < 0.05).

Two-way ANOVAs were conducted to examine the effects of participation medium and age on each of the estimated parameters (Figure 4). Across all five parameters, there were no significant interaction effects [α: *F*(3, 79) = 0.13, *p* = 0.72; β: *F*(3, 79) = 0.78, *p* = 0.38; weight: *F*(3, 79) = 0.013, *p* = 0.91; λ: *F*(3, 79) = 0.06, *p* = 0.82; perseveration: *F*(3, 79) = 0.63, *p* = 0.43]. Consistent with previous studies, we found a main effect of age group on the weight parameter [*F*(3, 79) = 31.81, *p* < 0.001], such that young adults were more goal-directed or model-based than older adults in both the in-person and online setting (in-person: *t* = 2.78, *df* = 20.97 *p* = 0.01; online: *t* = 4.90, *df* = 42.84, *p* < 0.001). We also found a main effect of age on β [*F*(3, 79) = 24.24, *p* < 0.001]. However, despite not showing a significant interaction effect of participation medium and age group, *post hoc* comparisons revealed only a significant difference between older and younger adults on β in online participation and not in-person participation, where older adults behaved more stochastically than young adults online (*t* = 5.03, *df* = 45.53, *p* < 0.001). There were no main effects of age group on α, λ, and perseveration.

We also performed ANCOVAs to observe the effect of participation medium, controlling for age. There were no significant interaction effects between participation medium and age (e.g., there was homogeneity of regression slopes), and the ANCOVA model was rerun excluding the interaction term. Age was a significant predictor of weight [β*_*age*_* = −0.002, *F*(2, 80) = 25.95, *p* < 0.001] and of β [β*_*age*_* = −0.07, *F*(2, 80) = 17.60, *p* < 0.001], such that greater age was related to more habitual decision-making and more stochastic responses. Age was also a significant predictor of perseveration [β*_*age*_* = 0.005, *F*(2, 80) = 4.08, *p* = 0.05], where greater age was related to more perseverative, or repetitive, responses. Age was not a significant predictor of α nor λ. Consistent with the ANOVAs, there were also no differences on any of the estimated parameters between in-person and online task participation after controlling for the effect of age [α: *F*(2, 80) = 0.0001, *p* = 0.99; β: *F*(2, 80) = 0.023, *p* = 0.98; weight: *F*(2, 80) = 0.47, *p* = 0.49; λ: *F*(2, 80) = 0.29, *p* = 0.59; perseveration: *F*(2, 80) = 0.38, *p* = 0.54].

## Discussion

Conducting a study online has many advantages, including larger sample sizes and being able to continue research even during a pandemic with restricted in-person activities. While the two-stage task has been conducted online in previous studies, to our knowledge, none of these have compared in-person to online performance on the two-stage task in older adults. This is critical because it is currently unclear whether high-quality decision-making data can be reliably collected via online task participation in older adults as previous findings have shown that older adults have more difficulties learning computer programs (White et al., 1999; Adams et al., 2005), and performing a task online may have greater attentional demands than in the lab setting.

In this study, we validated a web-based version of the two-stage decision task by simulating behavior on the models and successfully recovered the parameters. We also replicated behavioral results between in-person and online participation in young and older adults and found no differences across the estimated free parameters between in-person and online participation within the young adult group and the older adult groups. Most importantly, despite having a small sample of in-person participants, we replicated the primary effect of interest: we found more goal-directed decision making in young adults than older adults across both the in-person group (Eppinger et al., 2013; de Wit et al., 2014; Worthy et al., 2014) and the online groups. A recent study by da Silva and Hare (2020) found that improving instructions on the two-stage task lead to more goal-directed behavior. It is possible that more older adults were habitual performers in our study because they may have had more difficulty understanding the standard instructions used on the task. However, further work is needed to explore this. Nonetheless, to our knowledge, our study is the first to replicate this effect in an online study, although we note that Nussenbaum and colleagues also showed an age-effect of decision-making balance in children, adolescents, and young adults in an online version of the two-stage task (Nussenbaum et al., 2020). This suggests that the effect of age is quite robust and can be replicated both in-lab and online.

Interestingly, we found a significant difference in the parameter β between the young adult and older adult groups that participated in the study online, but we did not find this difference between the in-person groups. In the two-stage task, the β parameter corresponds to the stochasticity, or randomness, of choices, where β = 0 corresponds to completely random choices, and choices become more deterministic as β increases. One possible explanation for the disparate results between the online and in-person groups for the β parameter could have resulted from the technological demands of setting up the task at home. The older adults may have had a more difficult time with the online task as it requires more set up on their part, compared to in-person participants. Although all the older adults in this group were able to successfully complete the task, they may have already been more tired when they started the task as a result of having to navigate technology to set up remote desktop. However, in either of the online groups, there was no relationship between participants’ self-rating of computer usage at home to β (see Supplementary Material). We also analyzed the first and second half of trials separately in the older adults who participated online and found no differences between estimated parameters (see Supplementary Material). This suggests that fatigue did not play a role in driving more random responses. The most likely explanation is that the online group had more participants than the in-person group due to the shut-down of in-person data collection. If so, this effect may have been detected in the in-person group if we had a larger sample. Indeed, similar to the online group, the β parameter was higher in the in-person young adult group compared to the in-person older adult group, but this did not reach significance.

### Advantages and Disadvantages to Online Studies

Although the primary goal of our study was to determine whether estimated parameters in the online group in both young and older adults was comparable to those in-person, we also want to highlight some benefits and drawbacks from conducting a web-based study.

The advantages of running an online study are quite obvious: convenience and access to larger sample sizes. In a lab-based study, the pool of potential participants is limited by geographical constraints. An online study is limited insofar as the guidelines set by the IRB and/or funding sources allow. This can result in larger and more diverse, representative samples (Berinsky et al., 2012; Casler et al., 2013), although we acknowledge that we did not end up with a very racially diverse sample particularly in the online young adult group due to the use of convenience sampling. An online study can also be conducted from the participant’s home and remove the need to travel, making participation more accessible to individuals with physical disabilities or those who may have time constraints. For example, participating in a short study during their workday or at the end of the workday is more feasible without the need to travel for potential participants who work full time.

There are also a number of noteworthy drawbacks to running an online study that should be taken into consideration for future studies. First and perhaps most importantly, even though an online study removes the geographical barriers of participating in a study, participation is still constrained to those who have computer and reliable internet access. In our study, because we used remote desktop to support the two-stage task which had time constraints on responses, our study pool was even more limited as it required fairly high-speed internet. This limitation should not be downplayed—it highlights disparities in both access to participation and representation in research. Moving forward, it is important to think more deeply on methods to increase access, such as lending out equipment with limited data plans. Related to this issue of access is the environment in which online participants partake in the study. Whereas a lab environment is generally quiet (and admittedly lacking in ecological validity), some online participants may not be able to find a quiet space in their home to limit distractions. Additionally, even though our online study was moderated by an experimenter on the phone, there was no way to fully ensure that online participants were always paying attention during the duration of the study without an experimenter physically present. Both the diversity of environments among online participants and lack of physical presence of an experimenter could potentially result in noisier data.

Yet another important consideration to make while conducting an online study is whether the participants would have the computer proficiency to set up and complete the study. As mentioned above, an online experiment requires more set up on the part of the participant compared to an in-person study. We originally planned on instructing participants to download an app-based version of the two-stage task using MATLAB Runtime (MathWorks, MA, United States). However, we switched to using Google Remote Desktop to reduce the onus on the participants to set up the task. This unfortunately came at the cost of requiring high-speed internet for the study (≥60 mbps) and being able to accurately measure participant response latencies due to variability in internet connection speeds (see section “Limitations and Future Directions”).

### Limitations and Future Directions

Finally, we would like to acknowledge a few limitations specific to our study. First, there were significant demographic differences between the in-person and online groups in young adults, and we did not have a diverse sample. This was due to the mixed use of convenience sampling and recruitment through social media as a result of making quick adaptations in this study in response to the COVID-19 pandemic. Furthermore, all of the in-person participants completed the study before the onset of pandemic, whereas the online participants participated online as a direct result of restrictions due to the pandemic. Related to this, we also had a larger sample of online participants than in-person participants. Despite these differences between the in-person and online groups, however, we did not find differences across the estimated free parameters between in-person and online participation within the young adult group and the older adult groups, demonstrating the feasibility of conducting data collection on the two-stage task online for both groups. Another potential limitation of this study is that online two-stage task performance may be biased toward participants who have greater computer proficiency. As mentioned above, we found no differences between participants’ self-rating of computer usage at home to response stochasticity (β). However, our measure of computer usage was likely lacking in sensitivity, and the use of a standardized and more sensitive measure, as opposed to a self-rated percentage, may have revealed a bias effect of computer proficiency. Finally, as discussed above, we were unable to accurately measure response latencies on the online version of the two-stage task due to the use of Google Remote Desktop. In the future, the two-stage task could be implemented on a web server to more accurate measure choice response times, which could provide further insight on disparities in reaction times between habitual and goal-directed choices (Schweighofer et al., 2006; Keramati et al., 2011).

Overall, despite some limitations to online studies that require careful consideration, conducting a research study online has many advantages. Here, we found online performance on the two-stage task was comparable to performing the task in the lab for both young and older adults and also replicated previous findings that young adults are generally more goal-directed than older adults. Our results suggest that, despite being a fairly lengthy study requiring focus and attention, online administration of the two-stage task is feasible across both young and older adults.

## Data Availability Statement

The original contributions presented in the study are included in the article/Supplementary Material, further inquiries can be directed to the corresponding author/s.

## Ethics Statement

The studies involving human participants were reviewed and approved by IRB Administrator: Andrea Tlaseca, University of Southern California Institutional Review Board. The patients/participants provided their written informed consent to participate in this study.

## Author Contributions

KLI carried out the experiment, analyzed the data, and wrote the manuscript. LC implemented and tested the computational modeling parts of the study. RR recruited and ran participants for the study. BK implemented an early version of the task and computational modeling scripts. JM provided feedback on the conceptualization of an earlier version of the study and revised the manuscript. NS supervised computational modeling aspects of the study and revised the manuscript. S-LL supervised the project and revised the manuscript. All authors contributed to the article and approved the submitted version.

## Conflict of Interest

The authors declare that the research was conducted in the absence of any commercial or financial relationships that could be construed as a potential conflict of interest.

## Footnotes

1 https://remotedesktop.google.com/

2 http://cran.us.r-project.org
