# Supplementary material for: Validating Habitual and Goal-Directed Decision-Making Performance Online in Healthy Older Adults
Source: Front Aging Neurosci. 2021 Jun 29;13:702810. doi: 10.3389/fnagi.2021.702810 (PMC8276057; doi:10.3389/fnagi.2021.702810)
Supplement: Supplementary file 1 [file Data_Sheet_1.docx]

Supplementary Material

# Training protocol

# Due to the difficult nature of the Two-Stage Decision Task, participants were given extensive training before they were allowed to progress to the main task. The task was broken down into three parts, where each part started with written and audio instructions and were followed by a number of practice trials.

#
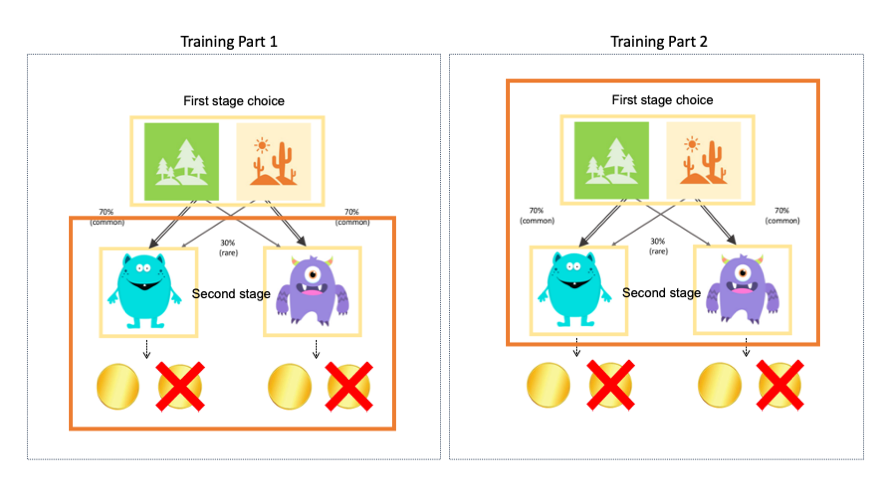
Supplementary Figure 1. Task Training. Part 1 of training focused on the association between the blue and purple cartoons to slowly changing reward probabilities. Part 2 focused on training participants on the 70-30 transition association between the location and the cartoons.

In the first part, participants were shown only the second stage cartoons and subsequent rewards (or no reward). Participants were told that the cartoon that just gave them a reward was likely to give a reward again. The goal was to train participants on the slowly changing reward probabilities associated with the second stage cartoons, and to train participants to respond to the reward. In this part of training, if the stay probability (that is, the overall percentage of times the participant chose the same choice as the previous trial) for reward trials was lower than the stay probability of unrewarded trials, participants were given feedback to select the cartoon that just gave them a coin and had to repeat this part of training again. While this particular feature likely contributes to a high learning rate (alpha), this was built in with the goal of ensuring that participants exhibited an effect of reward.

In the second part of training, participants were shown only the first stage locations (forest and desert) and the second stage cartoons. The goal of this second stage was to train participants on the association between the first and second stages. Participants were explicitly told about the 70%/30% transition structure between the two stages and given practice trials which exhibited this.

Finally, the third part of training combined the first and second parts, and participants were given practice trials on the full task. Following the third part of training, participants were given a comprehension quiz to ensure that they read and understood the instructions. If participants missed two or more questions, they were sent back to the beginning of training.

# Computational models of behavior

Choice behavior was fit according to the hybrid algorithm used in Gillan & Robbins, 2014, which itself is an adaptation from algorithms used in Daw et al., 2011 and Gläscher et al., 2010. Action values, or overall expected reward for an action, were first calculated for a pure model-based (goal-directed) learner and for a pure model-free (habitual) learner. These action values were then combined using a relative weighting free parameter *w*, which represents each individual’s propensity towards model-based versus model-free behavior, to obtain a hybrid learner action value.

## Model-free learner (habitual)

The first stage (s1) is composed of two actions (action a_a_ and action a_b_), which probabilistically leads to two second-stage states in stage 2 (s2_a_ and s2_b_). On trial t, the first-stage state is defined by s1_t_, the second-stage states by s2_t_, the chosen first-stage action by a_t_, and the second-stage rewards as r_t_.

The temporal difference learning algorithm is used for the model-free (mf) algorithm. The state-action value function, Q_mf_(state, action) is defined as follows for the first stage:

$Q_{mf}\left( s1\text{t+1}, a_{choice} \right)=Q_{mf}\left( s1\text{t+1}, a_{choice} \right)$ + $\alpha$ $*$ *ẟ_1,t_* + $\alpha*\lambda*$ *ẟ_2,t_*

where 𝛼 is a free learning rate parameter, which is assumed to be constant across the task for the individual; ẟ_1,t_ is the reward prediction error (RPE) at state 1, trial t; ẟ_2,t_ is the reward prediction error at stage 2, trial t and:

*ẟ_1,t_* = $Q_{mf}$(s2*_t_*) $-Q_{mf}$(s1, $a_{choice}$)

ẟ*_2,t_* = $reward$*_t_* $- Q_{mf}$(s2*_t_*)

such that the RPE for stage 1 is driven by the second-stage value, because the outcome of either reward or no reward, is only shown after stage 2. The first-stage value for the unselected action is devalued according to:

$$Q_{mf}\left( s1\text{t+1}, a_{unselected} \right)=\left( 1-\alpha\right)*Q_{mf}\left( s1,a_{unselected} \right)$$

And the values for the second stage are updated according to:

$Q_{mf}$(s2$\text{t+1}$) = $Q_{mf}$(s2*_t_*) + 𝛼 $*$ẟ*_2,t_*

## Model-based learner (goal-directed)

The pure model-based learner assigns values to actions by using an “internal model” of the task, here, the transition probabilities (which are explicitly communicated by the experimenter) in combination with information from the immediate reward values for each state.

Because immediate rewards are offered following the second stage, learning in the second stage is equivalent to that for the model-free learner. Hence *Q_mb_=Q_mf_* for the second stage states.

For the first stage, model-based values are defined as:

$Q_{mb}$($s1\text{t+1}, a\text{choice}$)= P(s2*_a_*| s1, $a_{choice}$) $* Q_{mf}$(s2*_a_*) + P(s2*_b_*| s1, $a_{choice}$) $*$ $Q_{mf}$(s2*_b_*)

where P(s2*_a_*| s1, $a_{choice}$) is the probability of ending up in state s2_a_ given stage 1 choice a_choice_ and P(s2*_b_*| s1, $a_{choice}$) is the probability of ending up in state s2_b_ given stage 1 choice a_choice_.

## Hybrid learner

For the hybrid learner, first stage choice actions are formed by a weighted average of the model-free and model-based learner, where the weight, w, is a free parameter. Q_Hybrid_(s1,a_choice_) is defined as:

$$Q_{hybrid}(s1,a_{choice}) = w*Q_{mb}(s1,a_{choice}) + (1-w)*Q_{mf}(s1,a_{choice})$$

## Choice rule

To connect action values to choices, the action values of the hybrid learner (Q_Hybrid_) were used as an input to the softmax equation to obtain the probability of each choice in stage 1 at each trial as:

$$P(a_{t+1}= a_{choice}|{s1}_{t}) =\frac{exp[\beta*Q_{hybrid}({s1}_{t},a_{choice})+p*rep(a_{choice})]}{\Sigma_{a^{'}}exp(\beta*Q_{hybrid}\left( {s1}_{t},a^{'} \right)*p+rep(a')}$$

where inverse temperature β represents the stochasticity of choices, perseveration p represents a stickiness parameter (staying with the same choice: p>0; switching: p<0), and rep(a) is defined as 1 if the same choice was made on the previous trial.

# No effects of self-rated computer usage on inverse temperature

We asked the online participants to self-rate how often they use computers from a scale of 0-100 (with 0 being not at all or never, 50 being sometimes, and 100 being extremely often). We then performed a regression model with the logit-transformed self-ratings on computer usage, group (young versus old). Only the main effect of group was significant (F = 12.98, p<0.0001), suggesting that self-rated computer usage was not a predictor in the consistency of responses.

# Analysis of first and second half of trials in online older adults

To examine whether fatigue played a role in causing more random responses in the older adults who participated in the study online (see Discussion), we separately estimated parameters in the first and second half of trials in this group. However, we found no differences in parameter estimates across all parameters, suggesting that fatigue did not play a role in causing more random responses.

**Supplementary Table 1.** **Comparison of first and second half of trials in online older adults**

| Parameter | Mean ± SD parameter estimate of trials 1-100 | Mean ± SD parameter estimate of trials 101-200 | t-value | p-value |
| --- | --- | --- | --- | --- |
| **α** | 0.65 ± 0.19 | 0.69 ± 0.21 | -0.70 | 0.43 |
| **β** | 3.49 ± 2.29 | 3.15 ± 1.82 | 0.63 | 0.53 |
| ***w*** | 0.38 ± 0.12 | 0.35 ± 0.13 | 0.93 | 0.36 |
| **λ** | 0.67 ± 0.15 | 0.72 ± 0.17 | -1.01 | 0.31 |
| ***ps*** | -0.18 ± 0.29 | -0.27 ± 0.38 | 1.00 | 0.32 |

# Parameter estimates for individual subjects


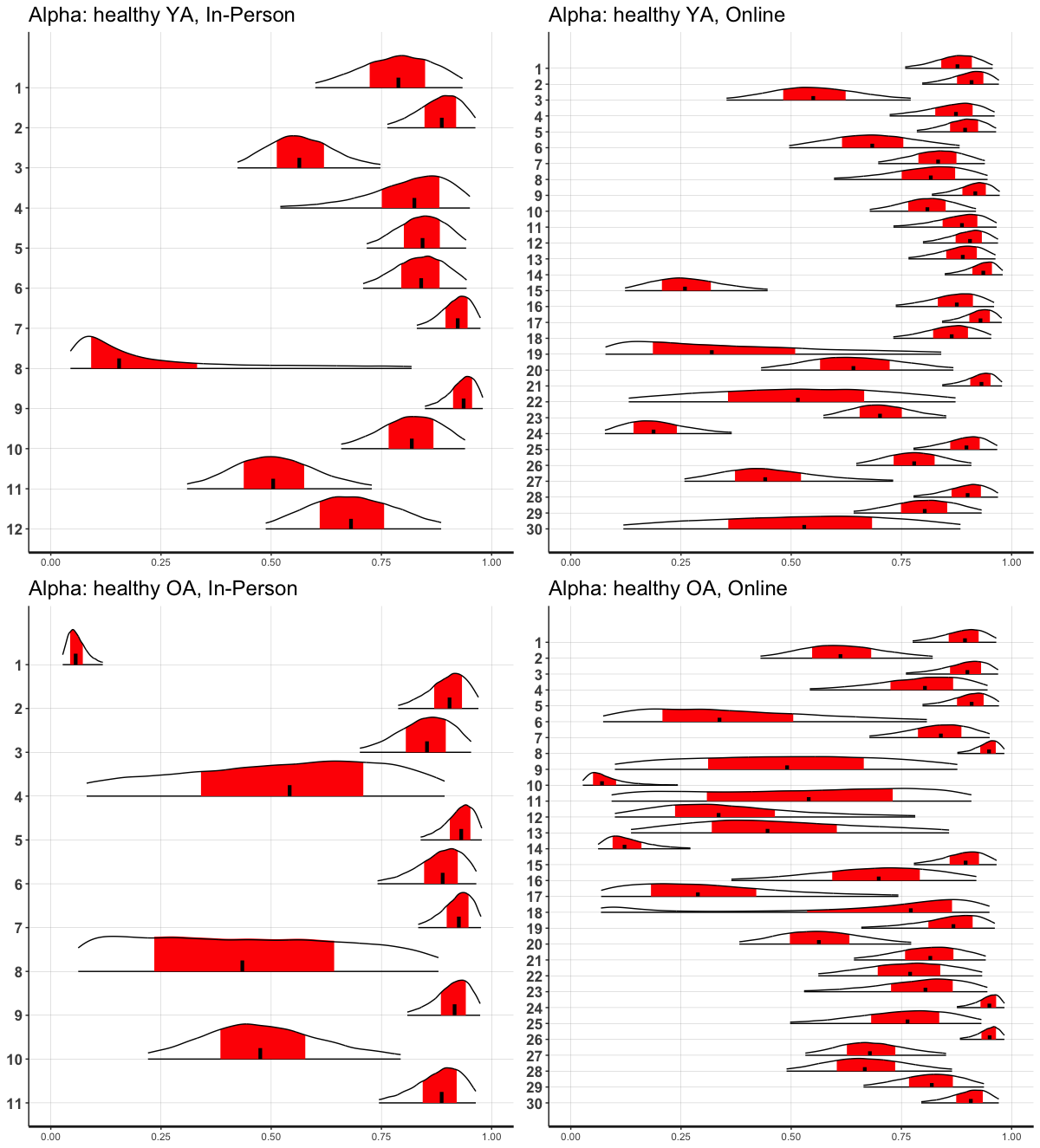


**Supplementary Figure 2. Alpha (learning rate)**


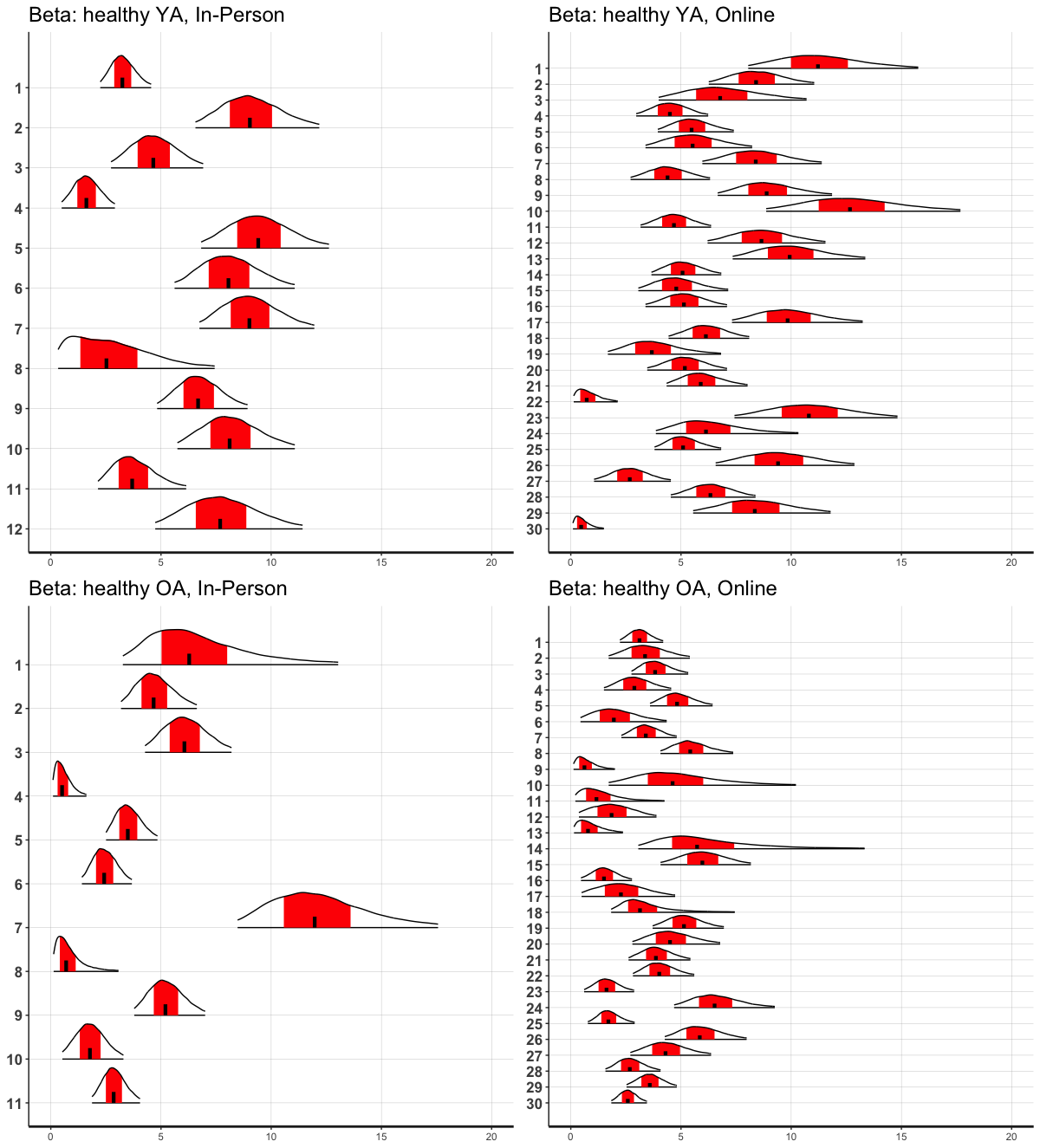


**Supplementary Figure 3. Beta (inverse temperature)**


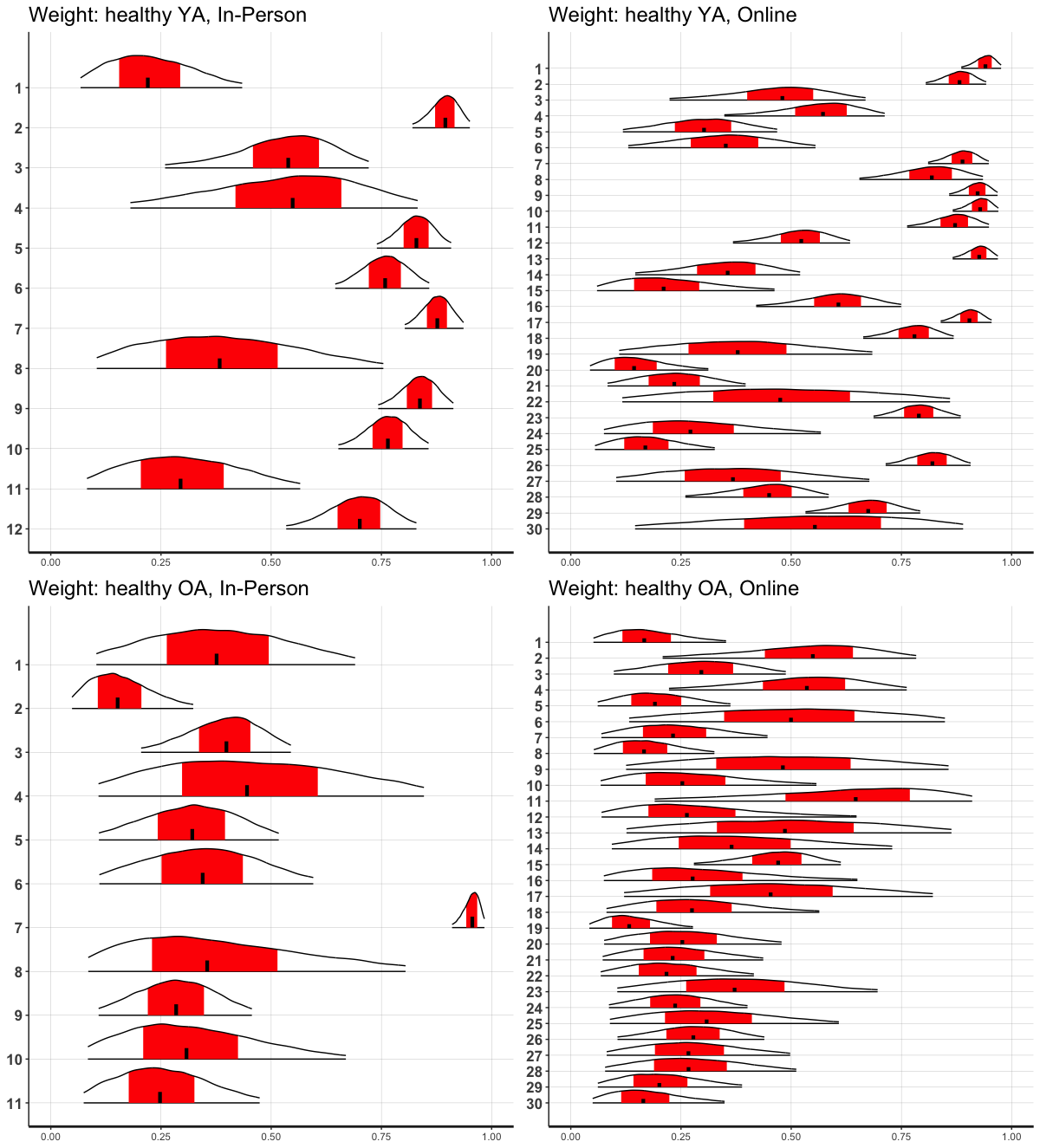


**Supplementary Figure 4. Weight**


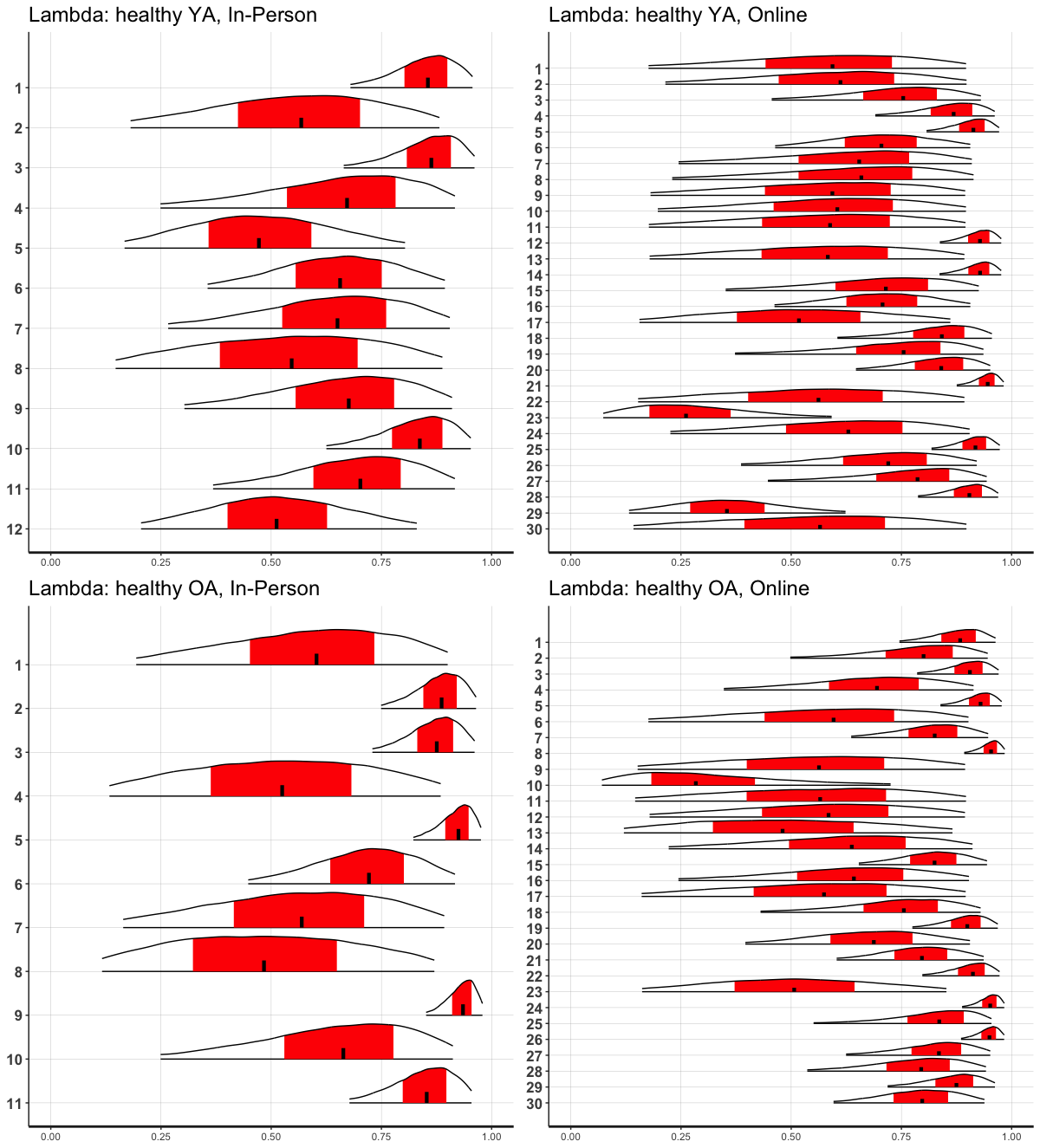


**Supplementary Figure 5. Lambda (eligibility trace)**


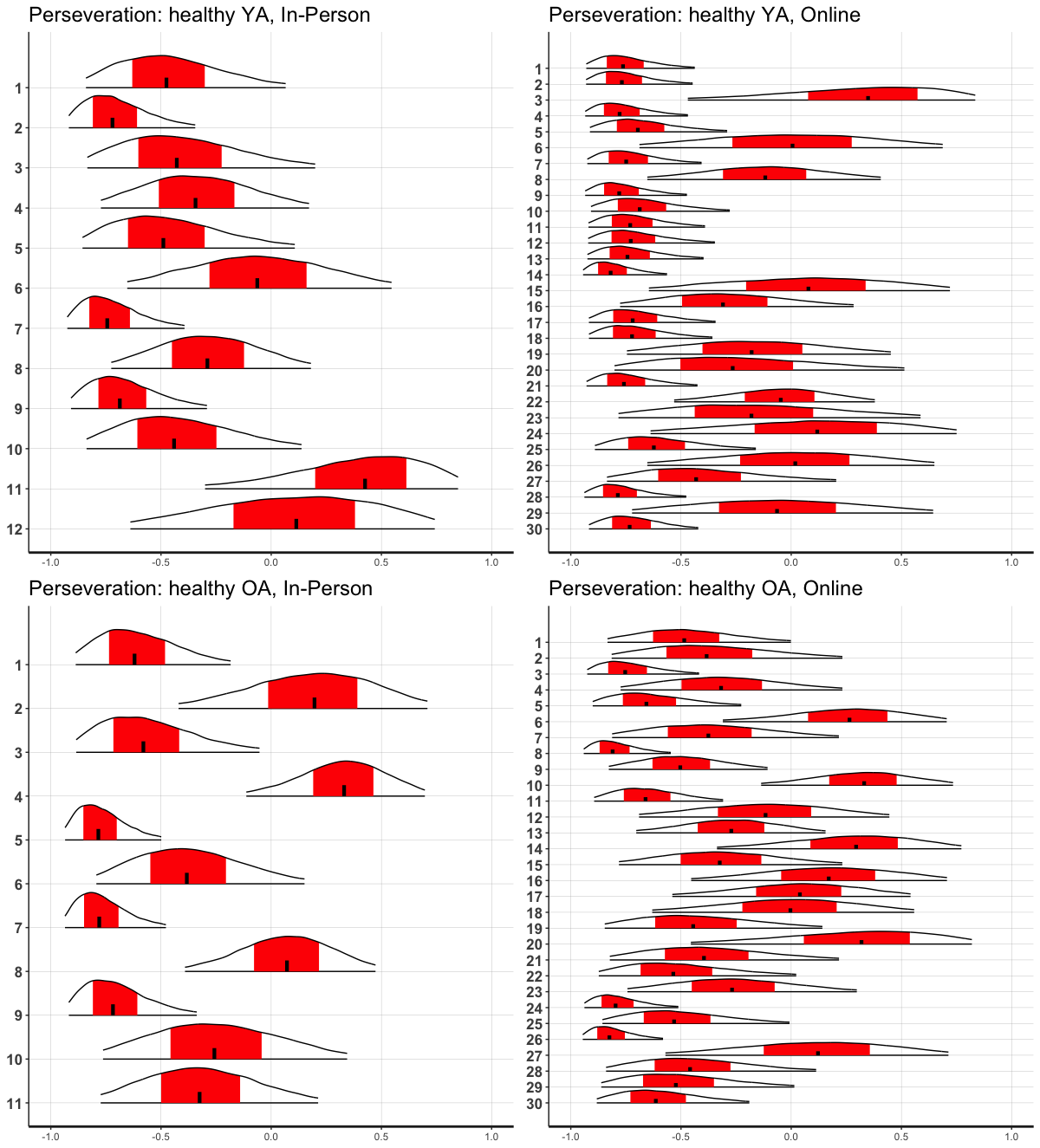


**Supplementary Figure 6. Perseveration**
